# Double-Outlet Right Ventricular Malformation in a Two-Year-Old Aberdeen Angus Cow

**DOI:** 10.3390/ani15172550

**Published:** 2025-08-30

**Authors:** Baker White, Francisco R. Carvallo-Chaigneau, Thomas E. Cecere, Harold Mckenzie, Giulio Menciotti, Sebastián G. Umaña Sedó

**Affiliations:** 1 Department of Biomedical Sciences and Pathobiology, Virginia-Maryland College of Veterinary Medicine, Virginia Polytechnic Institute and State University, Blacksburg, VA 24601, USA; bakerw8@vt.edu (B.W.); fcarvallo@vt.edu (F.R.C.-C.); tcecere@vt.edu (T.E.C.); 2 Department of Large Animal Clinical Sciences, Virginia-Maryland College of Veterinary Medicine, Virginia Polytechnic Institute and State University, Blacksburg, VA 24601, USA; hmckenzi@vt.edu; 3 Department of Small Animal Clinical Sciences, Virginia-Maryland College of Veterinary Medicine, Virginia Polytechnic Institute and State University, Blacksburg, VA 24601, USA; giuliom@vt.edu

**Keywords:** congenital, heart defect, pulmonary hypertension, ventricular septal defect, bovine

## Abstract

A 2-year-old Aberdeen Angus cow was presented with lethargy and decreased appetite at the VA-MD College of Veterinary Medicine Large Animal Teaching Hospital. Initial clinical examination revealed cyanosis, tachycardia, polycythemia, and a significant increase in lactate levels. The heifer experienced spontaneous death while hospitalized, prompting a postmortem examination. Gross evaluation demonstrated that the aorta arose entirely from the right ventricle, while the main pulmonary artery maintained its normal position, consistent with a diagnosis of double-outlet right ventricle. Additional cardiac abnormalities were identified, including an atrial septal defect, ventricular septal defect and marked right ventricular hypertrophy. These defects fall under the category of double-outlet right ventricle malformation. While congenital heart defects are a more recognized cause of cardiac failure and mortality in calves, they should remain a consideration in cases of sudden death, even in adult cattle.

## Simple Summary

Congenital heart defects are likely underdiagnosed in cattle, as postmortem examinations are not routinely performed in the field. When diagnosed, these defects are typically identified in calves, with much less recognition in adult cattle. This case report describes a 2-year-old Aberdeen Angus heifer diagnosed postmortem with a double-outlet right ventricle (DORV) approximately 2.5 days after arrival at the VA-MD College of Veterinary Medicine Large Animal Teaching Hospital. The objective of this report is to determine the cause of death based on clinical and pathological findings. These insights highlight the need to consider congenital cardiovascular anomalies in the differential diagnosis of adult cattle presenting with nonspecific clinical signs.

## 1. Background

Congenital heart defects can be potentially considered genetic in nature, but exact causes are often unknown, resulting in these defects being frequently regarded as developmental anomalies [1]. Other potential causes include environmental, toxicologic, infectious and nutritional factors, alone or in combination [2]. The published prevalence of congenital heart defects in cattle is low, between 0.2% and 2.7% [2]. However, the prevalence of congenital heart defects in cattle may be underestimated, as most animals are examined in field settings by large animal clinicians, where limitations in diagnostic resources and the absence of opportunities to perform a detailed cardiac auscultation may hinder accurate detection.

Congenital heart defect classification has been inconsistently presented throughout the existing literature, with multiple descriptors being utilized. A common categorization system classifies congenital heart defects as either cyanogenic or non-cyanogenic based on the presence or absence of mucous membrane cyanosis [2,3]. Congenital heart defects are also commonly categorized via a sequential segmental approach, which describes and identifies pathology within each individual morphologic structure. While these classification systems provide a useful framework, their utility depends on a confirmed diagnosis, which is not always possible in field conditions. Additionally, cases of congenital heart defects in cattle are often presented in advanced clinical stages, showing signs such as dyspnea, fever, recumbency, and abnormal respiratory sounds [4]. These clinical signs are not specific to heart failure and may also result from concurrent conditions such as respiratory disease, systemic infections, or metabolic disturbances, thus hindering the diagnosis of congenital heart defects.

The most frequently identified congenital defect in cattle is ventricular septal defect (VSD), with atrial septal defect (ASD) being the second most common [3]. Both uncomplicated (i.e., left to right shunting) VSD and ASD are non-cyanogenic defects, with cyanogenic defects such as Tetralogy of Fallot and Double-Outlet Right Ventricle (DORV) seen less frequently in cattle [2]. In addition, congenital heart defects cases are mostly diagnosed in young cattle, with hospital-based reports indicating an average diagnosis at 2 months of age, ranging from 1 to 11 months [4]. This report describes the case of a double-outlet right ventricle malformation in a 2-year-old Aberdeen Angus cow.

## 2. Case Presentation

A 2-year-old Angus heifer was referred to the Virginia–Maryland College of Veterinary Medicine (VA–MD CVM) for fever (40.5 °C), crackles in the right lung lobes, dark “black” blood, and petechia on mucous membranes. Prior to referral, the attending veterinarian administered 5 gallons of electrolyte solution via drench in response to a markedly elevated hematocrit of 81%. At the moment of arrival to the VA–MD CVM Large Animal Teaching Hospital the patient had a body condition score of 6 (i.e., 1 = emaciated; 9 = obese) and a body weight of 339.5 Kg.

A clinical examination conducted by the Production Management Medicine service veterinarian revealed a 2–4 mm eyeball recession indicating a mild dehydration level (6–8%) and a rectal temperature of 40.6 °C (Table 1). Heart rate was elevated at 120 bpm, and respiratory rate was also elevated at 80 bpm. Cardiothoracic auscultation revealed crackles in the right lung. No murmurs or arrhythmias were appreciated. Additional findings included darkened venous blood from the tail vein, petechiae on the vulvar mucous membranes, and hyperemic sclera and conjunctiva in both eyes were also present. Gastrointestinal examination revealed one weak ruminal contraction per minute and dry, scant feces. Ventral edema and peripheral venous distension were not detected during the physical examination. A stall-side blood work revealed a packed cell volume (PCV) of 81%, 6.2 g/dL of total protein (TP) and a lactate of 31.17 mg/dL (Table 1). A complete blook work revealed marked erythrocytosis and hemoconcentration with the following abnormalities: red blood cell count (RBC) of 14.74 × 10^6^ cells/µL, hemoglobin concentration 27.8 g/dL, hematocrit of 72.1%.

Thoracic ultrasonography identified multifocal to coalescing comet tail artifacts in the right and left cranial lung lobes. Following the initial assessment, the heifer was hospitalized for continuing monitoring and received intravenous hypertonic saline and a drench containing a mineral mix dissolved in 20 L of water with electrolyte solution to address hydration.

The patient was transferred to the VA-MD CVM Teaching Hospital Large Animal Internal Medicine service 12 h later for intensive monitoring and supportive care. On physical examination (Table 1), the patient was quiet and depressed. Her heart rate was severely elevated at 124 bpm, respiratory rate of 28 bpm with increased respiratory effort, and normal temperature of 39.2 °C. Her mucous membranes and sclera were hyperemic. There was a small amount of serous nasal discharge from both nostrils. Cardiothoracic auscultation revealed harsh lung sounds bilaterally. No murmurs or arrhythmias were appreciated. No rumen contractions were appreciated. An inconsistent soft ping over the right mid-abdomen was appreciated. Palpation per rectum revealed a doughy impaction of the rumen. When loose in her stall, the patient was standing with her head and neck extended, and obvious expiratory stridor was appreciated.

Repeat stall side blood-work revealed a PCV of 78%, TP of 9.0 g/dL, and a lactate level that was too high to read (Table 1). Complete blood count revealed severe polycythemia characterized by a total RBC of 14.74 × 10^6^ cells/uL, hemoglobin of 27.8 g/dL, hematocrit 72.1%, PCV 76.4%, mean corpuscular hemoglobin 18.9 pg/cell and a mean corpuscular hemoglobin concentration 38.6 g/dL (Table 2). Total white cell count was within the normal range at 10.23 × 10^3^ cells/uL, with mild mature neutrophilia (segmented neutrophils 4.5 × 10^3^ cells/uL). The patient also exhibited a moderate thrombocytopenia, with a platelet count of 53,000 cells/uL, and an elevated plasma protein of 9.7 g/dL (Table 2). The elevated lactate and heart rate in conjunction with polycythemia suggested poor tissue oxygenation.

An abdominal and thoracic ultrasound revealed multifocal to coalescing comet tail artifacts within all lung fields bilaterally, suggesting pleural surface abnormalities. Mild distension of the duodenum with decreased to no motility was also observed. An orogastric tube was placed, with freely flowing rumen fluid with an elevated chloride level observed.

**Figure 1:**
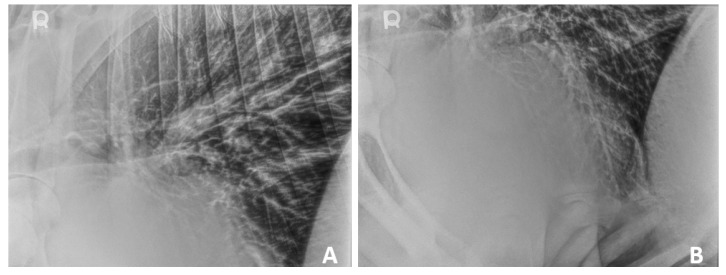
Standing thoracic radiography of a 2-year-old Aberdeen Angus with double-outlet right ventricular malformation. (**A**): Diffuse broncho-interstitial pattern throughout the lungs. (**B**): Diffuse broncho-interstitial pattern throughout the lungs, with a focal alveolar pattern caudoventrally to the apex of the heart.

Thoracic radiographs (standing left-to-right laterals) were obtained (Figure 1A,B). Thoracic imaging demonstrated a diffuse broncho-interstitial pattern throughout the lung fields which raised the suspicion for chronic bronchitis or diffuse bronchopneumonia. There was also a focal alveolar pattern caudoventral to the cardiac silhouette, which raised suspicion for mild aspiration pneumonia or a localized exacerbation of bronchopneumonia. There was no evidence of thoracic lymphadenopathy or pleural effusion.

A blood smear was performed, and basophilic, ring-shaped inclusion bodies were found, which were consistent with infection with *Babesia* spp. or *Theilieria* spp. An intravenous catheter was placed to initiate treatment, which included fluid therapy, oxytetracycline, flunixin meglumine, and dexamethasone. Oxygen therapy via an intranasal cannula was administered as well. Spontaneous death occurred in the hospital overnight within less than 48 h of being admitted. The carcass was submitted for necropsy to the Virginia Tech Animal Laboratory Services at VA—MD CVM.

**Figure 2:**
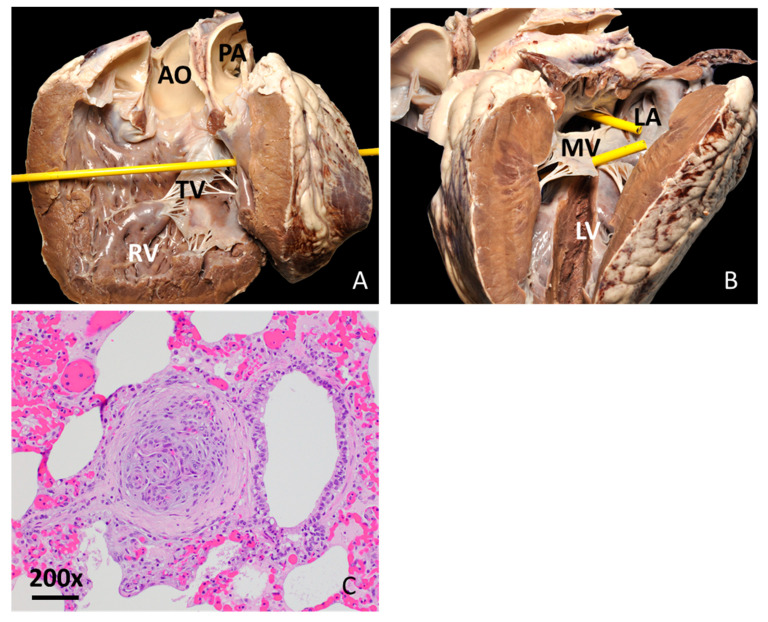
Heart post-mortem findings in a 2-year-old Aberdeen Angus cow with double-outlet right ventricular malformation. (**A**): Sagittal section through the right heart, viewed from a right lateral perspective. The right ventricular (RV) wall is markedly thickened. The aorta (AO) and pulmonary artery (PA) both stem from the RV. The yellow probe passes through perimembranous ventricular septal defect. TV: Tricuspid valve; AO: Aorta; PA: Pulmonary artery. (**B**): Sagittal section through the left heart viewed from a left lateral perspective. The yellow probes pass through septal defects: the upper probe passes through the secundum atrial septal defect in the interatrial septum, while the lower probe passes through a perimembranous ventricular septal defect in the interventricular septum. LV: Left ventricle; MV: Mitral valve; LA: Left atrium. (**C**): Lung, plexiform arterial proliferation of endothelial cells. Hematoxylin and eosin, 200×. Bar: 50 µm.

The autopsy revealed reddened ocular and gingival mucosae and diffuse violet discoloration and softening of all lung lobes. The heart was rounded and contained multifocal petechiae and ecchymoses along the epicardium. There were multiple cardiac malformations present. There was a secundum type ASD of 2.5 cm diameter in the atrial septum (Figure 2A). A perimembranous VSD of 1.9 cm diameter was also identified at the cranial aspect of the septal tricuspid valve leaflet (Figure 2B), along with a marked thickening of myocardium in the right ventricular wall. The ratio of thickness between the left and right ventricles was 1:1. The diameter of the proximal main pulmonary artery, measured at the right ventriculo-arterial junction, was 4.8 cm. Both the aorta and the main pulmonary artery arose solely from the right ventricle, with the aorta positioned to the right of the pulmonary artery. The diameter of the ascending aorta was 5.1 cm and displayed all three aortic leaflets and the openings for the coronary arteries (Figure 2A,B). The aortic leaflets presented fibrous continuity with both the tricuspid and the mitral valve. No patent ductus arteriosus was observed.

A fresh sample of the spleen was collected for *Theileria orientalis* and *Babesia* spp. PCR test which resulted positive for *Theileria orientalis*. Sections of the lung, liver, spleen, kidneys, adrenal glands, pancreas, rumen, abomasum and intestine were routinely processed and stained with hematoxylin and eosin stain for histopathology. Signs of pulmonary hypertension were noted in the lungs, characterized by multifocal plexiform lesions within the arteries adjacent to the bronchioles (Figure 2C). In other pulmonary vessels, there was formation of neointima, proliferation and thickening of the media, and narrowing of the vascular lumen. Small infiltrations with macrophages, lymphocytes and plasma cells were observed at the periphery of the bronchioles. In the liver, there was acute centrilobular degeneration and necrosis of hepatocytes, with mild neutrophilic infiltration. Findings in the liver were consistent with hypoxemia due to cardiac abnormalities and concurrent presence of *T. orientalis*.

## 3. Discussion

**Figure 3:**
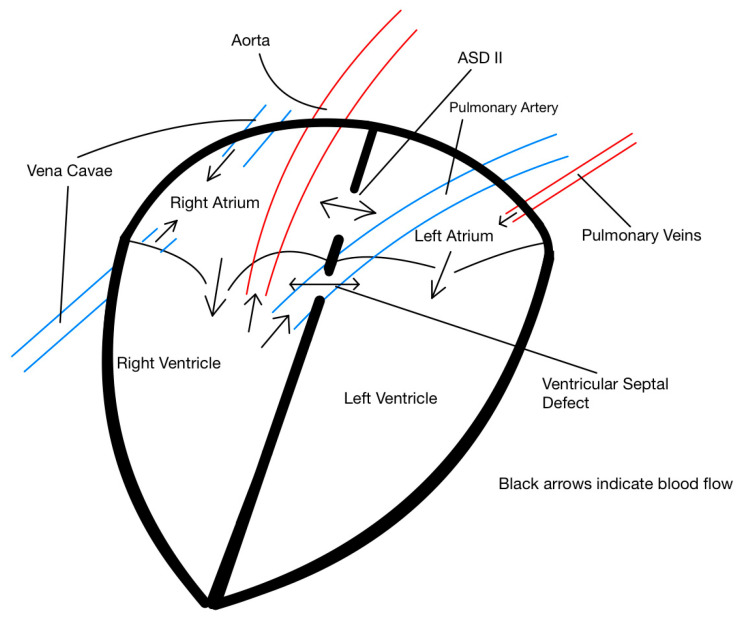
Schematic representation of intracardiac and great vessel anatomy illustrating abnormal blood flow patterns in a heart of a 2-year-old Aberdeen Angus cow with an atrial septal defect (ASD II) and a ventricular septal defect (VSD). The aorta and pulmonary artery both originate from the right ventricle, consistent with a double-outlet right ventricle (DORV). Black arrows indicate blood flow direction. Note the left-to-right shunting at the atrial level and the complex flow dynamics at the ventricular level due to the septal defects.

The cardiac abnormalities found in the post-mortem evaluation, namely the position of both the aorta and pulmonary artery arising solely from the right ventricle, can be grouped under the umbrella of cardiac DORV malformation, a complex congenital heart defect rarely reported in adult cattle (Figure 3; [4]). It is relevant to note that important diagnostics methods, such as echocardiogram and arterial blood gas analysis, were not conducted partly due to the financial constraints imposed by owner and the brief time the patient remained in the hospital after being admitted. This represents a significant limitation since both tests could have provided a definitive diagnosis. However, it must also be noted that echocardiography in adult cattle can be challenging, and adequate visualization of the heart may have not been possible. The reader should consider this when interpreting our assessment of the underlying pathophysiological processes.

In complex congenital heart diseases, the blood flow pattern established in utero tends to persist in postnatal life [5]. In DORV, the degree of blood flow directed to the aorta and pulmonary artery depends on numerous factors including the degree of dextroposition of the aorta, the partitioning of left ventricular output between pulmonary and systemic circulations, the relative systemic and pulmonary resistance, and the presence or absence of other cardiac anomalies [6]. Since this cow survived 2 years, we suspect that marked pulmonary hypertension early in life may not have been present. In the presence of the identified congenital anomalies, it is plausible that both ventricles operated under equal systemic pressures from an early age, potentially exposing the pulmonary vasculature to excessive flow and pressure. Such exposure may have contributed to progressive pulmonary hypertension and vascular remodeling. This may be supported by the results of the lung histology and possibly by the enlarged pulmonary artery observed at postmortem examination. In healthy adult Holstein Friesian and Jersey, the diameter of the pulmonary artery is typically 8–9 mm smaller than the ascending aorta [7], suggesting abnormal pulmonary vascular dilation in this case. However, the difference in diameter between the pulmonary artery and the aorta in the scientific literature is from dairy cattle breeds and not beef cattle like our case, which makes it difficult to compare our findings with published values.

Marked erythrocytosis in this patient, evidenced by a red blood cell count of 14.74 × 10^6^/µL, hemoglobin concentration of 27.8 g/dL, and hematocrit of 72.1%, is consistent with compensatory polycythemia, likely driven by chronic systemic hypoxemia. This is a physiological response mediated by increased erythropoietin secretion to stimulate red blood cell production enhancing oxygen-carrying capacity [8]. We considered that the polycythemia in this patient could have increased blood viscosity and exacerbate tissue hypoxia due to viscosity-induced increased flow resistance in the micro-vasculature, further decreasing capillary oxygen delivery. These possibilities highlight the complex pathophysiology of such congenital defects and the need for cautious interpretation in the absence of in vivo hemodynamic data.

It is important to highlight that the cow was infected with *Theileria orientalis*, a common tick-borne protozoan parasite in the Southeast United States. *Theileria orientalis* causes hemolysis, leading to anemia, hypoxia, and potentially multi-organ dysfunction [9]. In this case, tissue hypoxia is supported by the presence of hyperlactatemia observed in the blood analysis. However, the polycythemia associated with the DORV may have masked hematologic signs of active parasitemia and could have contributed to hypoxemia, making it difficult to definitively confirm *T. orientalis* as a clinically significant contributor to this patient.

Interestingly, no heart murmurs were detected during clinical examination despite the presence of congenital heart defects and the severity of clinical findings. The accuracy of cardiac auscultation in detecting structural heart disease varies widely among clinicians, with reported sensitivities ranging from 30% to 100% and specificities from 28% to 100% [10]. According to a systematic review by Davidsen et al. (2023) [10], the ability to detect heart murmurs is associated with clinician specialty; general practitioners were significantly less likely to recognize murmurs compared to cardiologists or internal medicine specialists. Therefore, it is plausible that the clinicians evaluating this patient may have lacked the specific experience or training required to detect subtle or low-grade cardiac murmurs. Also, it is important to consider that other factors such as the patient forelimb musculature and respiratory sounds could have decreased the sensitivity of the auscultation [11]. Additionally, the cardiac anatomy in this case may have contributed to the absence of an audible murmur. Large septal defects often produce lower velocity flow disturbances and therefore lower frequency, less audible murmurs, whereas smaller defects tend to create more pronounced high-velocity jets and harsher murmurs [12]. In this patient, the presence of large septal defects combined with potentially balanced pressures between the left and right ventricles, possibly due to concurrent pulmonary hypertension, could have minimized flow turbulence across the defects. However, we cannot confirm this hemodynamic scenario occurred in the absence of echocardiographic or intracardiac pressure measurements.

From a comparative perspective, DORV has been described in humans and in most domestic animal species. In humans, it is a rare disorder, comprising less than 1% of all congenital heart defects, and in more than 90% of cases, it occurs concurrently with other major heart abnormalities [13]. Following prenatal diagnosis of DORV via fetal echocardiography, most parents elect to terminate the pregnancy [13]. Surgical repair is frequently performed in human patients with a mean age of 1.9 to 2.1 years old. Although several surgical techniques are available, variations in biventricular repair are most commonly conducted to correct DORV in humans, depending on the specific anatomy of the defect [14]. The primary cause of early mortality in human surgical patients is preoperative pulmonary hypertension [14]. Ten-year survival in humans following surgical repair of DORV has been stated to be 89.5%, while the presence of atrioventricular septal defects increased mortality rate [15]. Research in humans regarding survival time in untreated DORV is minimal, and the defect is unlikely to be compatible with adult life in most cases.

DORV has also been described in multiple animal species, including dogs, cats [16], horses [17], and bovines [18]. While DORV is most reported in younger animals, there are a few cases of adult dogs and cats living with DOVR under medical management [16]. Clinical signs across species vary by individual, but tachypnea, mucous membrane cyanosis, exercise intolerance, delayed growth, polycythemia, lethargy, and recumbency are commonly reported. In that context, the clinical signs observed at presentation in this cow included cyanosis, tachypnea, lethargy, and markedly elevated PCV, which are consistent with reported clinical signs of bovines with congenital heart disease [4]. To the authors’ knowledge, however, there are no published reports of DORV in adult bovines. This case is therefore unique in that the cow reached full maturity before exhibiting overt signs of cardiac disease. While the defect ultimately proved incompatible with life, survival for two years is remarkable when compared to previously reported cases in large animals with similar congenital cardiac malformations.

## 4. Conclusions

We presented the case of a 2-year-old Aberdeen Angus cow patient with heart anomalies consistent with DORV and a secundum atrial septal defect, a condition typically diagnosed in animals at a younger age. While congenital heart defects are a more common differential in calves with heart failure symptoms, authors suggests that veterinarians include heart examination when investigating clinical signs associated with poor tissue oxygenation in individual adult cattle, especially in case of sudden death. We intend for this case presentation to improve diagnostic awareness and contribute to the limited epidemiological knowledge of heart conditions in cattle.

## Figures and Tables

**Table 1:** Results of the first and second clinical evaluation and stall-side bloodwork from a 2-year-old Aberdeen Angus cow with a double-outlet right ventricular malformation.

Parameter	First Result ^1^	Second Result ^2^	Reference Interval
Heart Rate	120 beats per minute	124 beats per minute	40–80 beats per minute
Respiratory Rate	80 breaths per minute	28 breaths per minute	12–36 breaths per minute
Temperature	38.3 °C	38.9 °C	37.7–39.2 °C
Packed Cell Volume	81%	78%	24–37%
Total Protein	6.2 g/dL	9.0 g/dL	6.7–8.8 g/dL
Lactate	31.17 mg/dL	Too high to read +	2.91–17.44 mg/dL

^1^ Exam conducted by the Production Management Medicine clinician at the moment of arrival. ^2^ Exam conducted by the Large Animal Internal Medicine Clinician after patient transfer.

**Table 2:** Second Hemogram of a 2-year-old Aberdeen Angus cow with a double-outlet right ventricular malformation.

Parameter	Result	Reference Interval
Total Red Blood Cells	14.74 × 10^6^ cells/µL	4.9–7.2 × 10^6^ cells/µL
Hemoglobin	27.8 g/dL	8.4–12.2 g/dL
Hematocrit	72.1%	23–33%
Mean Corpuscular Hemoglobin	18.9 pg/cell	14–18 pg/cell
Mean Corpuscular Hemoglobin Concentration	38.6 g/dL	36–39 g/dL
Total WBC Count	10.23 × 10^3^ cells/µL	5.8–12.6 × 10^3^ cells/µL
Segmented Neutrophils	4.5 × 10^3^ cells/µL	2.3–6.8 × 10^3^ cells/µL
Platelets	53 × 10^3^ cells/µL	233–690 × 10^3^ cells/µL
Plasma Protein	9.7 g/dL	6.5–8.5 g/dL
Packed Cell Volume	76.4%	24–37%

## Data Availability

The original contributions presented in this case report are included in the article. Further inquiries can be directed to the corresponding authors.

## Abbreviations

The following abbreviations are used in this manuscript:

ASD	Atrial septal defect.
VSD	Ventricular septal defect.
DORV	Double-outlet right ventricle.
PVC	Packed cell volume.
TP	Total protein.
